# Shark liver oil supplementation enriches endogenous plasmalogens and reduces markers of dyslipidemia and inflammation

**DOI:** 10.1016/j.jlr.2021.100092

**Published:** 2021-06-17

**Authors:** Sudip Paul, Adam Alexander T. Smith, Kevin Culham, Kevin A. Gunawan, Jacqueline M. Weir, Michelle A. Cinel, Kaushala S. Jayawardana, Natalie A. Mellett, Man K.S. Lee, Andrew J. Murphy, Graeme I. Lancaster, Paul J. Nestel, Bronwyn A. Kingwell, Peter J. Meikle

**Affiliations:** 1 Metabolomics Laboratory, Baker Heart and Diabetes Institute, Melbourne, Victoria, Australia; 2 Faculty of Medicine, Nursing and Health Sciences, Monash University, Melbourne, Victoria, Australia; 3 Haematopoiesis and Leukocyte Biology Laboratory, Baker Heart and Diabetes Institute, Melbourne, Victoria, Australia; 4 Metabolic and Vascular Physiology Laboratory, Baker Heart and Diabetes Institute, Melbourne, Victoria, Australia

**Keywords:** diet and dietary lipids, plasmalogens, lipidomics, lipid metabolism, inflammation, metabolic disease, immunometabolism, BH, Benjamini-Hochberg, COH, cholesterol, hsCRP, high-sensitivity C-reactive protein, LPC(O), lysoalkylphosphatidylcholine, PC, phosphatidylcholine, PC(O), alkyl phosphatidylcholine, PC(P), alkenyl phosphatidylcholine, PE(O), alkyl phosphatidylethanolamine, PE(P), alkenyl phosphatidylethanolamine, SLO, shark liver oil, TG(O), monoalkyl-diacylglycerol

## Abstract

Plasmalogens are membrane glycerophospholipids with diverse biological functions. Reduced plasmalogen levels have been observed in metabolic diseases; hence, increasing their levels might be beneficial in ameliorating these conditions. Shark liver oil (SLO) is a rich source of alkylglycerols that can be metabolized into plasmalogens. This study was designed to evaluate the impact of SLO supplementation on endogenous plasmalogen levels in individuals with features of metabolic disease. In this randomized, double-blind, placebo-controlled cross-over study, the participants (10 overweight or obese males) received 4-g Alkyrol® (purified SLO) or placebo (methylcellulose) per day for 3 weeks followed by a 3-week washout phase and were then crossed over to 3 weeks of the alternate placebo/Alkyrol® treatment. SLO supplementation led to significant changes in plasma and circulatory white blood cell lipidomes, notably increased levels of plasmalogens and other ether lipids. In addition, SLO supplementation significantly decreased the plasma levels of total free cholesterol, triglycerides, and C-reactive protein. These findings suggest that SLO supplementation can enrich plasma and cellular plasmalogens and this enrichment may provide protection against obesity-related dyslipidemia and inflammation.

Metabolic disease refers to a group of complex chronic conditions including obesity, type 2 diabetes, cardiovascular disease, and certain forms of cancer (1). These disorders share some common pathogenic features including altered lipid metabolism or dyslipidemia, which often leads to lipid accumulation at diverse cellular/tissue locations. Such aberrant lipid accumulation alters cell and/or tissue function, inducing events such as oxidative stress and inflammation that contribute to disease pathogenesis. Lipidomic profiling provides the opportunity to identify novel lipid signatures in metabolic diseases and explore their relationship with disease pathogenesis (2). Using this approach, a deficit of circulating plasmalogens has been identified as a feature of metabolic disease that is independent of age, sex, and BMI in multiple population and clinical cohorts (3, 4, 5, 6, 7).

Plasmalogens are a subclass of glycerophospholipids, primarily present as phosphatidylcholine (PC) and phosphatidylethanolamine (PE) species (8). They consist of a vinyl ether–linked fatty alcohol at the sn1 position and an acyl-linked fatty acid at the sn2 position of the glycerol backbone (Fig. 1C). Plasmalogens are often esterified with polyunsaturated fatty acids such as arachidonic acid (20:4) and docosahexaenoic acid (22:6), whereas the vinyl ether–linked residue is usually saturated (e.g., O-16:0 or O-18:0) or monounsaturated (O-18:1) (9).

**Fig. 1:**
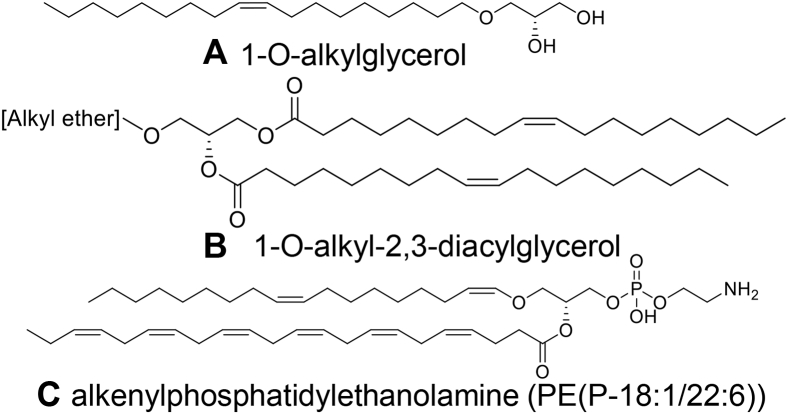
Structure of alkylglycerols and alkenyl phosphatidylethanolamine (plasmalogen). Alkylglycerols (A and B) are present in shark liver oil and can be metabolized into plasmalogens (C) in humans.

Plasmalogens have diverse biological functions. They are important constituents of the plasma membrane and can modulate its biophysical properties (10). They are also considered as endogenous antioxidants because of their vinyl ether linkage, which is highly susceptible to attack by reactive oxygen species, and this could therefore be helpful in protecting other biomolecules from oxidative damage (11, 12). In addition, plasmalogens may regulate cholesterol (COH) metabolism (13, 14) and immune responses (15, 16, 17, 18).

Plasmalogens are present in all mammalian tissues; however, their abundance varies across tissues and cell types such that levels are relatively high in the brain, heart, kidney, skeletal muscle, and certain immune cell types but lower in the liver and small intestine (9, 19). Plasmalogen biosynthesis involves a complex metabolic pathway through the peroxisome and endoplasmic reticulum (20) (Fig. 2). The rate-limiting steps occur in the peroxisome but can be bypassed through oral administration of alkylglycerols (Figs. 1A and 2). These alkylglycerols can be incorporated directly into the biosynthetic pathway (21) (Fig. 2) and lead to an increase in circulating and tissue plasmalogens (21, 22). Although alkylglycerols are present in our diet, the levels in typical western diets are insufficient to significantly boost our plasmalogen levels. Shark liver oil (SLO), a dietary supplement rich in alkylglycerols in the form of monoalkyl-diacylglycerols (TG(O)) (Fig. 1B), could be used to increase endogenous plasmalogen levels (Fig. 2). SLO has been used to treat a number of conditions, including lung inflammation (23), alimentary tract diseases (24), lymphadenopathy (25), cancer (26)s and dermatitis (27) and to help with wound healing (23). SLO supplementation also improved immune function in surgical patients (28). However, the mechanistic basis of the beneficial effects observed with SLO supplementation is not well defined, possibly because of the lack of proper understanding of the impact of SLO alkylglycerols on endogenous lipid metabolism.

**Fig. 2:**
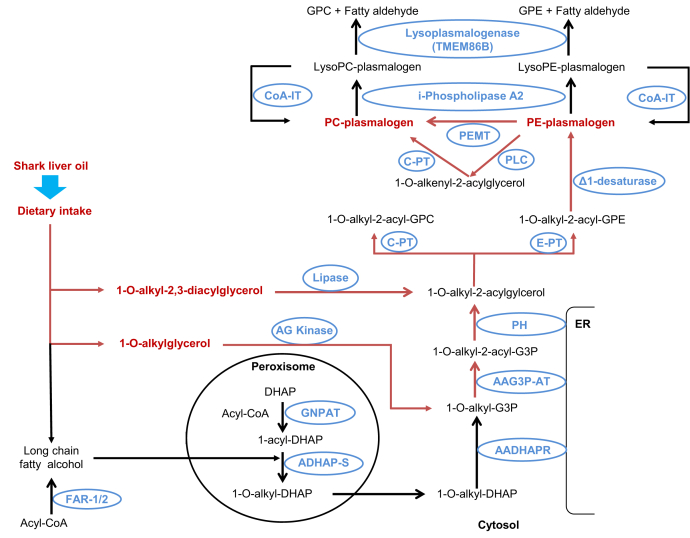
Plasmalogen biosynthesis and modulation by alkylglycerol precursors. Dietary alkylglycerols can bypass the rate-limiting peroxisomal biosynthetic steps (red pathway). Metabolites are shown in red and black and enzymes are shown in blue. AADHAP-R, alkyl/acyl-DHAP-reductase; AAG3P-AT, alkyl/acyl-glycero-3-phosphate acyltransferase; ADHAP-S, alkyl DHAP synthase; AG kinase, alkylglycerol kinase; CoA, coenzyme A; CoA-IT, coenzyme A–independent transacylase; C-PT, choline phosphotransferase; i-phospholipase A2, calcium-independent phospholipase A2; Δ1-desaturase, plasmanylethanolamine desaturase; DHAP, dihydroxyacetone phosphate; DHAP-AT, DHAP acyltransferase; E-PT, ethanolamine phosphotransferase; Far1/2, fatty acyl-CoA reductase 1 or 2; GPC, glycerophosphocholine; GPE, glycerophosphoethanolamine; PC, phosphatidylcholine; PE, phosphatidylethanolamine; PEMT, phosphatidylethanolamine N-methyltransferase; PH, phosphohydrolase; PLC, phospholipase C.

Here, we report on the characterization of the alkylglycerols contained within SLO and the effects of SLO supplementation on the plasma and cellular lipidome in overweight or obese individuals.

## Materials and methods

### Study design for SLO supplementation in humans

In this double-blind, placebo-controlled crossover study, participants (n=10) were overweight or obese (BMI in the range of 28–40 kg/m^2^) adult males (aged 25–60 years) with no signs of cardiovascular disease or diabetes. Among the 10 participants, only four met the definition of having the metabolic syndrome according to the strict International Diabetes Federation criteria (29); however, all the participants had at least two features of metabolic syndrome. Written informed consent was obtained from all study participants before the commencement of the study. This study was performed in accordance with the ethical principles set forth in the Declaration of Helsinki and received approval from the Alfred Hospital Ethics Committee (approval number: 436/15).

**Fig. 3:**
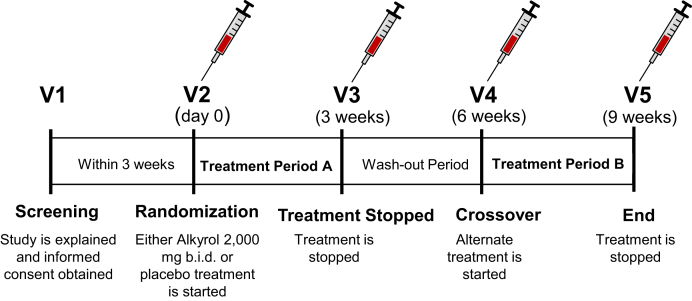
Study design for shark liver oil supplementation in humans. Participants were recruited into the study and asked to attend an initial screening. At the screening visit, participants underwent a medical examination to assess their eligibility. Eligible participants were recalled, within three weeks, where they were randomized to take either Alkyrol® (shark liver oil gel caps) or placebo for three weeks. At the three-week visit, the participants discontinued the treatment/placebo for a three-week washout period. At visit 4, the participants commenced the alternative treatment for 3 weeks. At visit 5, the participants underwent the same medical examination as visit 1 to assess any change throughout the study period. Fasting blood samples from each participant were collected at the initial screening and at the start and end of each intervention.

Participants were randomized into placebo or treatment arms and received 4-g Alkyrol® (purified SLO; Eurohealth, Ireland) per day or placebo (methylcellulose) for 3 weeks followed by a 3-week washout phase and were then crossed over to 3 weeks of the alternate placebo/Alkyrol® treatment. Methylcellulose was chosen as a placebo to avoid possible confounding effects of an oil-based placebo. Both Alkyrol® and methylcellulose capsules were prepared to have similar visual appearance. Participants were instructed to keep their dietary composition and food intake constant during the two treatment phases. Fasting blood samples were collected at the start and end of each intervention (Fig. 3).

### Isolation of plasma and white blood cells from whole blood

Participants’ blood samples were collected in K3-EDTA tubes and centrifuged at 1,711 *g* for 15 min at room temperature. The top plasma layer was aspirated, 1 μl of 100 mM butylhydroxytoluene per milliliter of plasma was added, and the plasma was stored at −80°C. The buffy layer was mixed with 8 ml of PBS and layered on top of 5 ml of Ficoll-Paque and centrifuged at 400 *g* for 30 min at room temperature with the lowest brake. The resulting upper layer (containing plasma and platelets) was discarded, and the thin cloudy layer of white blood cells was collected and transferred to a fresh tube. PBS (8 ml) was added, and the sample was centrifuged at 250 *g* for 10 min at room temperature with the highest brake. The cells were then resuspended in 1.5 ml of PBS and centrifuged at 100 *g* for 10 min at room temperature. After centrifugation, the supernatant was discarded and the white blood cell pellet was suspended in 400 μl PBS and stored at −80°C.

### Clinical measurements

The fasting plasma levels of glucose, COH, triglycerides, HDL-C, LDL-C, insulin, and high-sensitivity C-reactive protein (hsCRP) were measured using commercially available kits on a COBAS Integra 400 Plus blood chemistry analyzer (Roche Diagnostics, Australia) following standard procedures. Remnant COH was estimated as the total COH minus LDL-C minus HDL-C, non–HLD-C was calculated as total COH minus HDL-C, and homeostatic model assessment for insulin resistance was calculated as fasting insulin (mIU/l) multiplied by fasting glucose (mmol/l) and then divided by 22.5. The measurement of tumor necrosis factor alpha, monocyte chemoattractant protein-1, and vascular cell adhesion protein 1 levels in plasma was performed by Cardinal Bioresearch, Queensland, Australia.

### Complete blood count and flow cytometry

Complete blood count was performed on a Sysmex XS-1000i automated haematology analyser following manufacturer’s guidelines. Monocyte subpopulations (classical (CD14^++^CD16^−^), intermediate (CD14^+^CD16^+^), and nonclassical (CD14^dim^CD16^++^)) were identified by staining peripheral blood cells with anti-human antibodies (BD Pharmingen, San Diego, CA) specific for CD56 (MY31, PE), CD2 (RPA-2.10, PE), CD19 (HIB19, PE), NKp46 (9E2/NKp46, PE), CD15 (W6D3, PE), HLA-DR (TU36, FITC), CD14 (M5E2, Pacific Blue), and CD16 (3G8, PE-CY7). Briefly, 100 μl of whole blood was added to 5 ml of the lysis buffer (BD Pharm Lyse) and then incubated in the dark for 5 min. The sample was then added to 10 ml of the wash buffer (9:1 ratio of PBS and fetal bovine serum) and centrifuged at 300 *g* for 5 min at 4°C. The resulting pellet was then resuspended in the wash buffer, placed in an Eppendorf tube, and centrifuged at (300 *g*, 5 min, room temperature). Antibodies were then added to the samples and incubated for 30 min in the dark. The samples were washed with PBS and centrifuged (300 *g*, 5 min, room temperature) before transferring the cells to a fluorescence activated cell sorting tube. Finally, the cells were analyzed using the BD FACSCanto II flow cytometer. The following gating strategy was used to define the various monocyte populations: white blood cells were initially gated based on size and granularity (forward scatter and side scatter). To identify monocytes, cells were gated on the basis of being HLA-DR^+^ and cell-linage marker (CD56, CD2, CD19, NKp46, and CD15) negative. HLA-DR^+^ cells were subsequently assessed for CD14 and CD16 expression, with classical monocytes being defined as CD14^++^CD16^−^, intermediate monocytes being defined as CD14^+^CD16^+^, and nonclassical monocytes being defined as CD14^dim^CD16^++^, as described previously (30). The flow cytometry data were analyzed using the BD FACSDiva software.

### Lipidomic analysis

#### Characterization of TG(O) species in SLO

Alkyrol® was diluted 1:50,000 in chloroform:methanol (1:1) and infused into a QTRAP 4000 triple quadrupole mass spectrometer (AB Sciex) using a Harvard syringe pump at a flow rate of 20 μl/min, and a Q1 scan in positive-ion mode (mass range: 300–1,000 Da) was performed. The most abundant molecular species in SLO were then identified based on the peak intensity from the Q1 spectrum. For relative quantification of these species, Alkyrol® was diluted 10,000 times in chloroform:methanol (1:1) and 10 μl of diluted Alkyrol® was then mixed with 10 μl of internal standard mix (supplemental Table S1) and 40 μl of water-saturated butanol and 40 μl of methanol with 10 mM ammonium formate. The resultant mixture was then analyzed using the method described in the LC/MS/MS section.

#### Characterization of alkylglycerol composition in SLO

Alkylglycerols are present in Alkyrol® as TG(O), that is, consisting of one alkyl chain at the sn1 position and two acyl chains at sn2 and sn3 positions. The 1-O-alkylglycerol composition of Alkyrol® was determined after an alkaline hydrolysis of the acyl chains. In brief, Alkyrol® was diluted 10,000 times with chloroform:methanol (1:1) and 10 μl of the diluted samples was mixed with 100 μl of 0.8 M sodium hydroxide in methanol and then incubated at 37°C for 2 h. Then, 10 μl of 8 M formic acid was added to stop the hydrolysis reaction. Next, 10 μl of internal standard mix (supplemental Table S1) was added, and lipids were extracted following Folch extraction procedure (31) and finally reconstituted with 50 μl of water-saturated butanol and 50 μl of methanol with 10 mM ammonium formate. The extract was then analyzed using the method described in the LC/MS/MS section.

#### Extraction of lipids from plasma and white blood cells

Lipids were extracted using a single-phase chloroform:methanol (2:1) extraction protocol as described previously (5). Briefly, 10 μl of plasma or 20 μl of white blood cell pellet (suspended in PBS) was combined with 20 volumes (200 or 400 μl) of chloroform:methanol (2:1) and 10 μl of the internal standard mix (supplemental Table S1) and then vortexed. Samples were mixed in a rotary mixer for 10 min, sonicated for 30 min, and then allowed to stand for 20 min at room temperature. Samples were then centrifuged (16,000 *g*, 10 min, 20°C), and the supernatant was dried under a stream of nitrogen at 40^o^C. The extracted lipids were finally resuspended with 50 μl of water-saturated butanol and 50 μl of methanol containing 10 mM ammonium formate.

#### LC/MS/MS

Analysis of lipids were performed on an Agilent 1200 HPLC system coupled to an AB Sciex QTRAP 4000 triple quadrupole mass spectrometer using scheduled multiple reaction monitoring experiments described previously (32). LC separation was performed on a 2.1 × 100 mm C18 Poroshell column (Agilent) at 400 μl/min. The following gradient conditions were used: 10% B to 55% B over 3 min, then to 70% B over 8 min, to 89% B over 0.1 min, and finally to 100% B over 3.3 min. The solvent was then held at 100% B for 1 min. Equilibration was as follows: the solvent was decreased from 100% B to 10% B over 0.1 min and held for an additional 4.5 min. The solvent system consisted of solvent A: 50% water/30% acetonitrile/20% isopropanol (v/v/v) containing 10 mM ammonium formate and solvent B: 1% water/9% acetonitrile/90% isopropanol (v/v/v) containing 10 mM ammonium formate. The conditions for the MS/MS of each lipid class are provided in supplemental Table S1.

The concentrations of individual lipid species were calculated by taking a ratio of the area under the curve of the lipid of interest to the area under the curve of the internal standard of the corresponding lipid class (supplemental Table S1) and then multiplying the said ratio by the amount of internal standard added into the sample. Response factors were also applied for some lipid species (supplemental Table S2) to better estimate true lipid concentrations as described previously (6). Lipid class concentrations were calculated from the sum of individual species within that class. TG(O) species were measured both as single ion monitoring and neutral loss of specific fatty acyl/alkyl chains. As single ion monitoring measurements captured more diverse species, they were used for calculation of the concentration of total TG(O).

### Statistical analysis

Lipidomics data were either used as concentrations or as concentrations normalized to the total PC concentration. Zero values (i.e., values below the detection limit) and values more than 4.5 standard deviations below the mean of the considered lipid (i.e., extreme low outliers due to measurement errors around the detection limit) were set to missing. Values were log10-transformed before analyses. All missing values were then single-imputed using sample-wise k- nearest neighbor imputation (using k=5, given that only 10 participants were available at each time point). Modeling results for the lipids with imputed values may thus be considered as overconfident, although these results aligned well with results for other species in these classes.

For each lipid species or class as well as for clinical measures, blood cell count, monocyte subpopulations, and inflammatory markers, we posited linear mixed models explaining (log10) levels by an overall intercept, a treatment effect (either none or placebo/SLO at visits V3 and V5), and a carryover effect (either none or placebo/SLO at visit V4 only), with a random intercept for each participant. Contrasts for treatment and carryover effects were designed with across-group averaging vectors for use in the type-III ANOVAs below. The treatment contrast then compared placebo with baseline and SLO with placebo, whereas the carryover contrast compared placebo with none and SLO with none. For each species or total, the inclusion or exclusion of treatment or carryover effects was done by an ad hoc forward stepwise feature selection process: first, treatment was considered, and only included if the type-III ANOVA *P* value for that term was below 0.10, and then, carryover was considered in a similar way. Our modeling thus allowed carryover effects to be estimated independently of treatment effects. We allowed this as we reasoned that not all long-term effects (ie, at visit V4) of supplementation would necessarily reflect the direct effect of supplementation seen at visit V3: longer response times (ie, time taken for changes to be visible in the lipidome greater than the 3 weeks between visits V2 and V3), compensatory mechanisms, slow metabolism modifications, behavioral changes, and more might impact any carryover effect in a way unrelated to the treatment effect seen at V3. The similar pre-SLO and pre-placebo plasma ether lipid levels in the participants (supplemental Fig. S1) irrespective of their SLO treatment order (first or second) indicate that the 3-week washout period was sufficient.

We then extracted the beta coefficients, 95% confidence intervals, and corresponding post hoc *P* values from each model. We applied Benjamini-Hochberg (BH) multiple testing correction to said *P* values (because of the selection process above, the impact of the multiple testing correction was thus reduced for model terms that were less frequently included) across lipid species and classes separately. As the outcome was on a log scale, beta coefficients (and their confidence intervals) were transformed into fold changes by a power transformation (fc=10^beta^).

The mean percentage change of the alkenyl chain composition of plasma alkenyl phosphatidylethanolamine (PE plasmalogen or PE(P)) after Alkyrol® and placebo treatments were compared with repeated measures ANOVA using the combined data from the two intervention arms (visit 2 to 3 and visit 4 to 5), taking into account treatment (as a between-subject variable) and treatment order.

Corrected *P* values less than 0.05 were considered statistically significant. All analyses were performed in R (v3.5), in particular using packages lme4 (v1.1.21) and lmerTest (3.1.0) for the linear mixed modeling.

## Results

### Composition of alkylglycerols in SLO

**Fig. 4:**
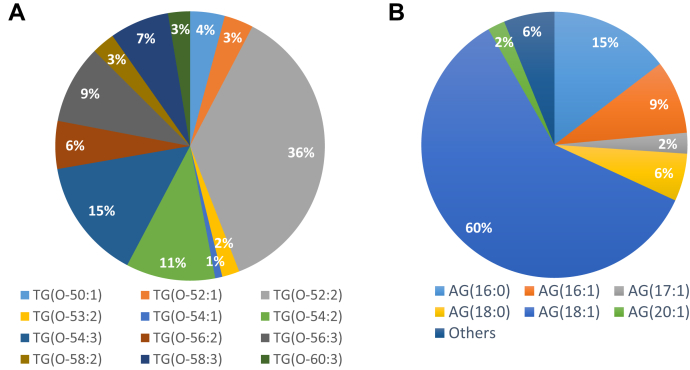
Alkylglycerol composition of shark liver oil. The relative proportions of (A) monoalkyl-diacylglycerol (TG(O)) species and (B) 1-O-alkylglycerols (AG) in the shark liver oil used in this study (Alkyrol®).

The relative proportions of major TG(O) species in Alkyrol® are presented in Fig. 4A and supplemental Table S3. The major TG(O) species in Alkyrol® are TG(O-52:2) (36%), TG(O-54:3) (15%), TG(O-54:2) (11%), TG(O-56:3) (9%), and TG(O-56:2) (6%). Neutral loss scans of different 1-O-alkyl and acyl chains provided us with possible sn1/sn2/sn3 compositions of these TG(O) species (supplemental Table S3). The results also depict that the 1-O-alkyl portion of these TG(O) species is dominated by the O-18:1 alkyl chain, whereas the fatty acyl portion is more diverse and mostly consisting of 16:0, 18;1 20:1, 22:1, and 24:1 acyl chains (supplemental Table S3). Quantitative analysis of the 1-O-alkyl chain portion of the TG(O) species (after saponification of the acyl chains) showed 60% as the O-18:1 alkyl chain. The other abundant 1-O-alkyl chains were O-16:0, O-16:1, O-17:1, O-18:0, and O-20:1 (Fig. 4B and supplemental Table S4).

### Baseline characteristics of the participant cohort

This study consisted of 10 male participants and was conducted between December 2015 and August 2016. Table 1 presents the baseline characteristics of the participants. The average age and BMI of the participants were 50 years and 32 kg/m^2^, respectively. The blood pressure, heart rate, and biochemical parameters of the participants were within the normal range. No side effects were reported for any participants when treated with SLO.

**Table 1:** Baseline characteristics of the participants

Characteristics^a^	Mean ± Standard Deviation
Age (years)	50 ± 10
BMI (kg/m^2^)	32.1 ± 3.2
Waist circumference (cm)	105.7 ± 9.4
Waist/hip ratio	0.96 ± 0.05
Systolic blood pressure (mmHg)	116 ± 13
Diastolic blood pressure (mmHg)	74 ± 10
Heart rate (bpm)	57 ± 8
Total cholesterol (mmol/l)	5.39 ± 1.19
High density lipoprotein cholesterol (mmol/l)	1.09 ± 0.12
Low density lipoprotein cholesterol (mmol/l)	3.32 ± 0.86
Triglycerides (mmol/l)	2.14 ± 1.08
Fasting plasma glucose (mmol/l)	4.90 ± 0.48

a n = 10 male participants

### The effect of SLO supplementation on the plasma lipidome

**Fig. 5:**
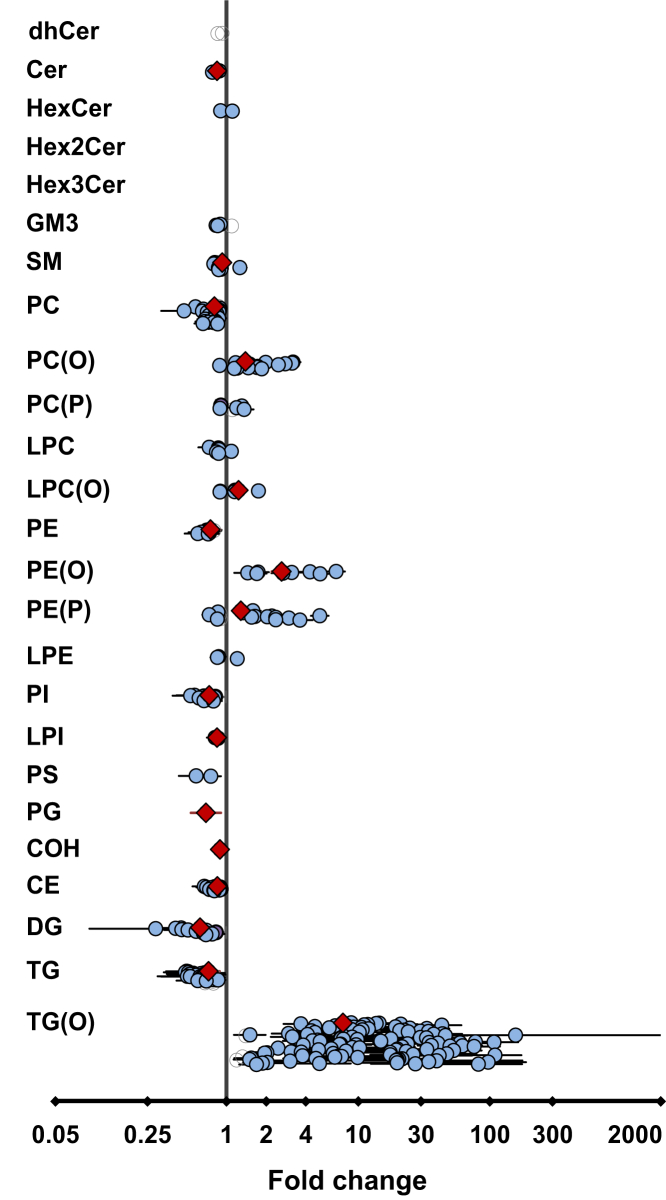
Effect of shark liver oil supplementation on plasma lipids. Estimated effects of shark liver oil (SLO) supplementation on (log-transformed) plasma lipid concentrations relative to placebo treatments. Open gray circles: lipid species, nonsignificant, no confidence intervals (CIs); violet circles: lipid species, nominally significant (*P* < 0.05), with CIs; blue circles: lipid species, significant after multiple testing correction (*P* < 0.05) using Benjamini-Hochberg’s approach, with CIs; red diamonds: lipid class/subclass totals, significant after multiple testing correction (*P* < 0.05) using Benjamini-Hochberg’s approach, with CIs. CE, cholesteryl ester; Cer, ceramide; COH, cholesterol; DG, diacylglycerol; dhCer, dihydroceramide; HexCer, monohexosylceramide; Hex2Cer, dihexosylceramide; Hex3Cer, trihexosylceramide; GM3, G_M3_ ganglioside; PC, phosphatidylcholine; PC(O), alkyl phosphatidylcholine; PC(P), alkenyl phosphatidylcholine; LPC, lysophosphatidylcholine; LPC(O), lysoalkylphosphatidylcholine; LPE, lysophosphatidylethanolamine; LPI, lysophosphatidylinositol; PE, phosphatidylethanolamine; PE(O), alkyl phosphatidylethanolamine; PE(P), alkenyl phosphatidylethanolamine; PG, phosphatidylglycerol; PI, phosphatidylinositol; PS, phosphatidylserine; SM, sphingomyelin; TG, triacylglycerol; TG(O), TG(O) [NL], monoalkyl-diacylglycerol.

We observed significant changes in 16 plasma lipid classes/subclasses (BH-corrected *P* < 0.05, Fig. 5 and supplemental Table S5) after SLO supplementation relative to placebo treatment. Among these, the levels of 5 lipid classes/subclasses were increased and 11 decreased with SLO supplementation. At a species level, the changes in 293 individual plasma lipid species after SLO treatment were significant (BH-corrected *P* < 0.05; Fig. 5 and supplemental Table S6). Of these, the levels of 139 lipid species were significantly decreased and the levels of 154 species were significantly increased. Of the 293 lipid species, 159 were ether lipids and 134 were nonether lipids.

**Fig. 6:**
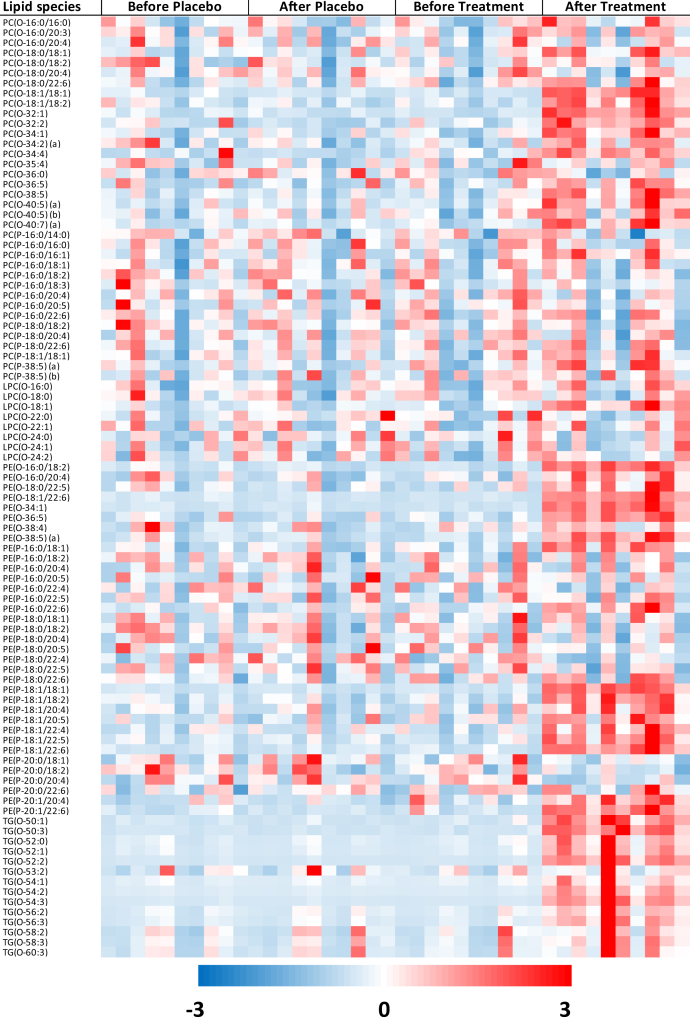
Effects of shark liver oil supplementation on plasma ether lipid species. Heat map of Z-scored log-transformed ether lipid concentrations in plasma. LPC(O), lysoalkylphosphatidylcholine; PC(O), alkyl phosphatidylcholine; PC(P), alkenyl phosphatidylcholine; PE(O), alkyl phosphatidylethanolamine; PE(P), alkenyl phosphatidylethanolamine; TG(O), TG(O) [SIM], monoalkyl-diacylglycerol.

As expected, there was a significant increase in total TG(O) level in the SLO treatment group compared with the placebo group (Fig. 5), which was because of increases in multiple TG(O) species (Fig. 6). We then compared the relative proportions of major TG(O) species in plasma of participants before and after SLO supplementation, and observed that the proportions of TG(O) species with medium-chain fatty acids (16–18 carbons) such as TG(O-52:1), TG(O-53:2), and TG(O-54:2) were mostly higher, whereas the proportions of species with comparatively longer chain fatty acids (20–24 carbons) such as TG(O-56:2), TG(O-56:3), TG(O-58:2), TG(O-58:3), and TG(O-60:3) were lower in the postsupplementation plasma relative to SLO (supplemental Fig. S2).

In addition to changes in TG(O), the plasma levels of ether phospholipids, alkyl phosphatidylcholine (PC(O)), lysoalkylphosphatidylcholine (LPC(O)), alkyl phosphatidylethanolamine (PE(O)), and PE(P) also increased significantly in the SLO treatment group compared with the placebo group (Fig. 5 and supplemental Table S5). When we looked into individual species, we observed that most of the PC(O) species were significantly elevated after SLO supplementation, although the greatest increase was observed for a species with a O-18:1 alkyl chain, PC(O-18:1/18:1) (fold change: 3.20, BH-corrected *P* = 9.98e-16; Fig. 6 and supplemental Table S6). Furthermore, we noted that the increase in the LPC(O-18:1) level was the highest among the increases in LPC(O) species (fold change: 1.75, BH-corrected *P* = 6.69e-12; Fig. 6 and supplemental Table S6). Similarly, in the case of PE(O) species, the increases were much greater for species with O-18:1 alkyl chain such as PE(O-34:1) and PE(O-18:1/22:6) than increases in other species (fold change: 6.82 and 5.13, respectively, BH-corrected *P* = 3.24e-19 and 3.62e-18, respectively; Fig. 6 and supplemental Table S6).

In contrast to these increases, there were significant decreases in ceramide, sphingomyelin, PC, PE, phosphatidylinositol, lysophosphatidylinositol, and phosphatidylglycerol levels after SLO treatment (Fig. 5 and supplemental Table S5). Moreover, there were significant reductions in the levels of COH, cholesterol ester, diacylglycerol, and triacylglycerol with SLO supplementation (Fig. 5 and supplemental Table S5).

Interestingly, it was also noted that the level of PC decreased significantly (Fig. 5 and supplemental Table S5) in the SLO treatment group. As PC is the major phospholipid making up the surface layer of all lipoprotein particles, this suggests either a decrease in the number of lipoprotein particles or a change in the surface lipid of the lipoprotein particles. To assess the relative change in lipid classes, we normalized the lipid data to total PC and performed the analysis on the normalized data. After normalization, 17 lipid classes/subclasses showed a significant difference between the responses to SLO relative to placebo (supplemental Fig. S3). Of interest, the increase in plasmalogen and other ether phospholipids is even more notable after accounting for decreasing total PC level, indicating a strong enrichment of these lipid species in the surface layer of the lipoprotein particles.

### The effect of SLO supplementation on molecular PE(P) species and PE(P) alkenyl chain composition in plasma

**Fig. 7:**
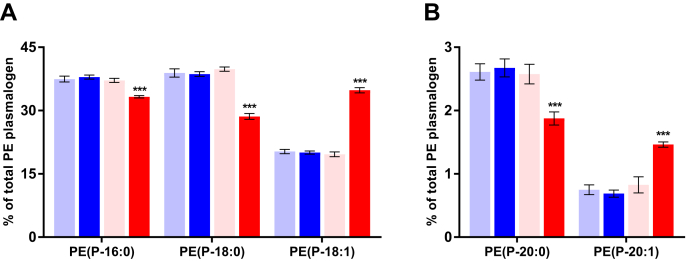
Changes in alkenyl chain composition of alkenyl phosphatidylethanolamine species after shark liver oil supplementation. Bars represent the mean relative abundance (whiskers represent ± standard error of mean) of (A) P-16:0, P-18:0, and P-18:1 and (B) P-20:0 and P-20:1 alkenyl chains in the alkenyl phosphatidylethanolamine [PE(P)] species before and after placebo or SLO treatment (before placebo: pale blue bar; after placebo: dark blue bar; before treatment: pale red bar and after treatment: dark red bar), irrespective of the intervention order. The nominal significance of the treatment effect was determined using repeated measures ANOVA; ∗∗∗ indicates *P* < 0.001. PE(P), alkenyl phosphatidylethanolamine; SLO, shark liver oil.

We observed that SLO supplementation predominantly increased the levels of PE(P) species with an 18:1 alkenyl chain (Fig. 6 and supplemental Table S6). Looking more closely into the relative abundance of PE plasmalogens with 5 different alkenyl chains available in this study (Fig. 7), we found that the increase in PE(P-18:1) (+72%, from ~20% to ~35%) came at the expense of PE(P-16:0) (-11%), PE(P-18:0) (-28%), and PE(P-20:0) (-26%). Interestingly, the levels of PE(P-20:1), although low, also seem to increase after SLO supplementation (+87%, from ~0.75% to ~1.5%).

### The effect of SLO supplementation on the white blood cell lipidome

**Fig. 8:**
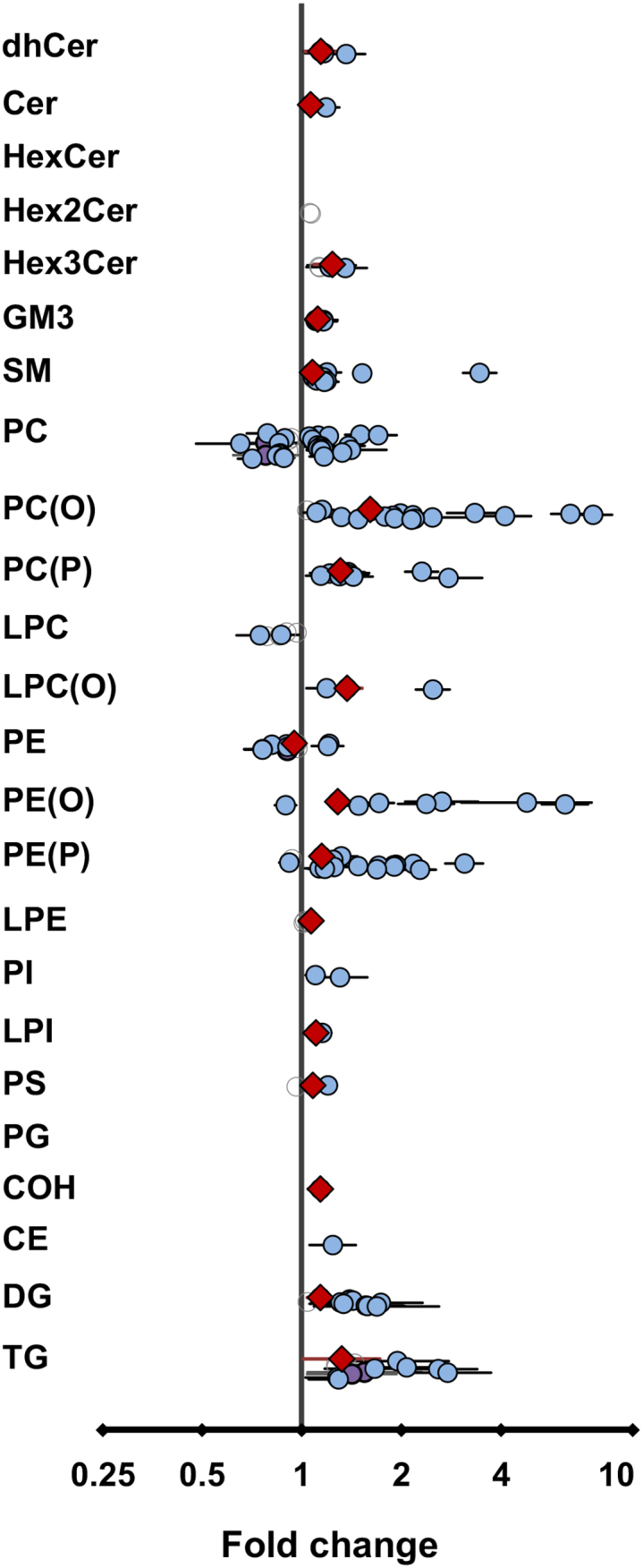
Effect of shark liver oil supplementation on white blood cell lipids. Estimated effects of shark liver oil (SLO) supplementation on (log-transformed) circulatory white blood cell lipid concentrations (normalized to phosphatidylcholine concentration) relative to placebo treatments. Open gray circles: lipid species, nonsignificant, no confidence intervals (CIs); violet circles: lipid species, nominally significant (*P* < 0.05), with CIs; blue circles: lipid species, significant after multiple testing correction (*P* < 0.05) using Benjamini-Hochberg’s approach, with CIs; red diamonds: lipid class/subclass totals, significant after multiple testing correction (*P* < 0.05) using Benjamini-Hochberg’s approach, with CIs. Cer, ceramide; COH, cholesterol; CdhCer, dihydroceramide; DG, diacylglycerol; E, cholesteryl ester; HexCer, monohexosylceramide; Hex2Cer, dihexosylceramide; Hex3Cer, trihexosylceramide; GM3, G_M3_ ganglioside; PC(O), alkyl phosphatidylcholine; PC(P), alkenyl phosphatidylcholine; LPC, lysophosphatidylcholine; LPC(O), lysoalkylphosphatidylcholine; LPE, lysophosphatidylethanolamine; LPI, lysophosphatidylinositol; PE, phosphatidylethanolamine; PE(O), alkyl phosphatidylethanolamine; PE(P), alkenyl phosphatidylethanolamine; PG, phosphatidylglycerol; PI, phosphatidylinositol; PS, phosphatidylserine; SM, sphingomyelin; TG, triacylglycerol.

Any potential beneficial effects of SLO supplementation on metabolic disease would likely be mediated by their uptake and metabolism in various cells and tissues. Accordingly, after demonstrating that SLO supplementation enriched the plasma lipidome with plasmalogens and other ether lipids, we wanted to determine if SLO supplementation could alter plasmalogen and ether lipids within cells. To do this, we isolated white blood cells from circulation and analyzed their lipidome. Indeed, SLO supplementation resulted in significant post-treatment changes in 14 lipid class/subclasses (after BH correction) in circulatory white blood cells (Fig. 8, supplemental Table S7). There were significant increases in the concentrations of ether phospholipids such as PC(O), alkenyl phosphatidylcholine (PC(P)), LPC(O), PE(O), and PE(P) with SLO supplementation. We observed that there were significant changes (after BH correction) in 131 lipid species after SLO supplementation (increases in 111 species and decreases in 20 species). For ether phospholipid species, increases were most noticeable for the species with O-18:1 alkyl/alkenyl chain (supplemental Table S8). In addition to the increases in ether phospholipids, we also observed significant increases in COH, sphingolipid, and other phospholipid and glycerolipid classes/subclasses after SLO supplementation (Fig. 8 and supplemental Table S7).

### The effect of SLO supplementation on clinical measures, inflammatory markers, blood cell counts, and monocyte populations

Although the present study was not explicitly designed to test the impact of SLO supplementation on clinical indices of metabolic dysfunction, we nonetheless thought it would be of interest to use the current experiment to conduct a pilot analysis to examine the potential of SLO supplementation to impact several clinical measures. We observed that there were significant decreases in the levels of total COH (BH-corrected *P* = 2.84e-03), non-HDL COH (BH-corrected *P* = 7.13e-03), and triglycerides (BH-corrected *P* = 4.84e-02) after SLO supplementation (Table 2). However, there were no significant changes in the plasma levels of fasting glucose, glycated hemoglobin, insulin, homeostatic model assessment for insulin resistance, HDL-C, LDL-C, and remnant COH with SLO treatment relative to placebo treatment (Table 2).

**Table 2:** Effect of shark liver oil supplementation on clinical measures, inflammatory markers, blood cell counts, and monocyte populations

Parameter	Before Placebo^a^	After Placebo^a^	Before SLO^a^	After SLO	Fold Change^b^	*P* value^c^	BH-Corrected *P* Value^d^
FPG (mmol/l)	5.01 ± 0.12	5.02 ± 0.15	5.10 ± 0.16	5.08 ± 0.17			
HbA1c (%)	5.48 ± 0.06	5.48 ± 0.09	5.46 ± 0.05	5.47 ± 0.09			
Insulin (mIU/l)	11.24 ± 1.17	10.30 ± 1.17	11.28 ± 1.73	9.64 ± 1.04			
HOMA-IR	2.50 ± 0.27	2.33 ± 0.30	2.58 ± 0.42	2.21 ± 0.28			
Cholesterol (mmol/l)	5.35 ± 0.34	5.49 ± 0.35	5.36 ± 0.34	4.98 ± 0.36	0.31	**2.58e-04**	**2.84e-03**
HDL-C (mmol/l)	1.17 ± 0.06	1.20 ± 0.06	1.13 ± 0.05	1.14 ± 0.06			
LDL-C (mmol/l)	3.26 ± 0.26	3.29 ± 0.22	3.20 ± 0.23	3.08 ± 0.25			
Non-HDL cholesterol (mmol/l)	4.18 ± 0.33	4.29 ± 0.34	4.23 ± 0.36	3.84 ± 0.35	0.35	**1.30e-03**	**7.13e-03**
Remnant cholesterol (mmol/l)	0.98 ± 0.15	1.06 ± 0.19	1.11 ± 0.23	0.88 ± 0.20			
Triglyceride (mmol/l)	2.01 ± 0.28	2.18 ± 0.38	2.25 ± 0.39	1.64 ± 0.35	0.29	**1.32e-02**	**4.84e-02**
hsCRP (mg/l)	1.86 ± 0.29	2.20 ± 0.42	2.70 ± 0.49	1.62 ± 0.17	0.26	**4.74e-02**	6.52e-02
TNFα (pg/ml)	3.38 ± 0.45	3.16 ± 0.30	3.52 ± 0.47	3.27 ± 0.53			
MCP-1 (pg/ml)	148.57 ± 9.23	146.07 ± 8.52	142.55 ± 10.28	156.48 ± 11.93			
VCAM-1 (ng/ml)	926.16 ± 72.99	881.66 ± 74.18	867.43 ± 83.42	870.04 ± 81.14			
Hb (g/l)	150 ± 2.46	149 ± 2.42	148 ± 2.37	145 ± 1.96	0.42	**4.27e-02**	6.52e-02
WBC (10^9^/l)	6.05 ± 0.26	6.43 ± 0.51	6.45 ± 0.35	5.82 ± 0.37	0.24	**3.70e-02**	6.52e-02
Platelets (10^9^/l)	212 ± 12.74	211 ± 10.96	213 ± 12.27	201 ± 8.83			
RBC (10^12^/l)	4.91 ± 0.10	4.87 ± 0.10	4.83 ± 0.10	4.71 ± 0.08	0.71	**2.39e-02**	5.27e-02
Hct (l/l)	0.45 ± 0.01	0.44 ± 0.01	0.44 ± 0.01	0.43 ± 0.00			
MCV (fl)	91.10 ± 0.80	90.50 ± 0.99	90.70 ± 0.92	90.90 ± 0.82			
MCH (pg)	30.50 ± 0.27	30.70 ± 0.21	30.70 ± 0.21	30.90 ± 0.23			
MCHC (g/l)	337.10 ± 1.29	338.70 ± 2.44	338.20 ± 2.41	338.80 ± 2.05			
RDW (%)	13.09 ± 0.17	13.21 ± 0.19	13.35 ± 0.18	13.13 ± 0.20			
Neutrophils (10^9^/l)	3.38 ± 0.19	3.78 ± 0.38	3.65 ± 0.29	3.26 ± 0.31	0.31	**1.76e-02**	**4.85e-02**
Lymphocytes (10^9^/l)	1.99 ± 0.11	1.92 ± 0.12	2.03 ± 0.13	1.90 ± 0.09			
Eosinophils (10^9^/l)	0.15 ± 0.01	0.17 ± 0.02	0.16 ± 0.02	0.15 ± 0.01			
Basophils (10^9^/l)	0.04 ± 0.00	0.04 ± 0.00	0.05 ± 0.01	0.04 ± 0.00			
Monocytes (10^9^/l)	0.49 ± 0.03	0.48 ± 0.06	0.57 ± 0.04	0.47 ± 0.02			
Classical monocytes (10^9^/l)	0.36 ± 0.04	0.32 ± 0.05	0.40 ± 0.05	0.35 ± 0.04			
Intermediate monocytes (10^9^/l)	0.02 ± 0.003	0.03 ± 0.005	0.043 ± 0.009	0.02 ± 0.003			
Non-classical monocytes (10^9^/l)	0.11 ± 0.01	0.13 ± 0.03	0.13 ± 0.02	0.11 ± 0.02			
Classical monocytes (%)	72.78 ± 3.23	66.63 ± 5.83	66.93 ± 6.29	71.46 ± 5.56			
Intermediate monocytes (%)	4.23 ± 0.85	5.82 ± 0.68	7.09 ± 1.43	4.26 ± 0.63			
Nonclassical monocytes (%)	23.02 ± 3.29	27.55 ± 6.22	25.68 ± 6.91	25.63 ± 5.54			

FPG, fasting plasma glucose; Hb, hemoglobin; HbA1c, glycated hemoglobin; Hct, hematocrit; HOMA-IR, homeostatic model assessment for insulin resistance; HDL-C, high density lipoprotein cholesterol; hsCRP, high sensitive c-reactive protein; LDL-C, low density lipoprotein cholesterol; MCH, mean corpuscular hemoglobin; MCHC, mean corpuscular hemoglobin concentration; MCP-1, monocyte chemoattractant protein-1; MCV, mean corpuscular volume; RBC, red blood cell; RDW, red cell distribution width; TNFα, tumor necrosis factor alpha; VCAM-1, vascular cell adhesion protein 1; WBC, white blood cell.

a Data are presented as the mean ± SEM.

b Fold change of SLO treatment effect.

c *P* values for estimated treatment effects obtained from a linear mixed model.

d Benjamini-Hochberg–corrected *P* values for estimated treatment effects.

SLO supplementation did reduce the level of hsCRP relative to placebo treatment (BH-corrected *P* = 6.82e-02, Table 2). However, we note that the hsCRP level was higher in the pre-SLO group than pre-placebo group with a higher standard error, suggesting that acute effects in some individuals may have contributed to this result. There was no significant effect of SLO supplementation on other inflammatory cytokines (tumor necrosis factor alpha, monocyte chemoattractant protein-1, and vascular cell adhesion protein 1) (Table 2).

There was a borderline significant decrease in the total number of white blood cells after SLO supplementation (BH-corrected *P* = 6.52e-02, Table 2). This was mostly due to a trend to the reduction in the number of neutrophils after SLO treatment (BH-corrected *P* = 4.85e-02, Table 2). In addition, there were decreases in hemoglobin (Hb) and red blood cell (RBC) levels with SLO supplementation (BH-corrected *P* = 6.52e-02 and 5.27e-02, respectively, Table 2). SLO supplementation did not show any significant effect on the other measures of whole blood count (Table 2). We also evaluated the impact of SLO supplementation on total circulatory monocyte count and monocyte subsets but did not observe any significant effect of SLO supplementation on monocyte populations (Table 2).

## Discussion

SLO has long been used as a traditional dietary supplement for therapeutic health benefits in many countries (33, 34). One of the most active ingredients in SLO is the alkylglycerols (35); however, the impact of alkylglycerols on endogenous lipids in humans has not previously been reported. In this study, 4 g per day of SLO supplementation in overweight or obese individuals over a three-week period resulted in a significant increase in circulating levels of multiple ether lipid classes including PC(O), LPC(O), PE(O), PE(P), and TG(O). Importantly, SLO supplementation also led to a significant increase in the levels of plasmalogens and other ether phospholipids within white blood cells. Although further large trials with SLO supplementation will be required, our results provide evidence that SLO supplementation may have clinical utility in patients with metabolic disease. More specifically, we observed reductions in the levels of free COH (BH-corrected *P* = 2.84e-03), triglycerides (BH-corrected *P* = 4.84e-02), and hsCRP (BH-corrected *P* = 6.52e-02) with SLO supplementation.

### SLO supplementation impacts the plasma lipidome

We observed a substantial increase in plasma TG(O) (665%) level after SLO supplementation. Considering the low TG(O) concentration in human plasma and the high concentrations of TG(O) in SLO, this is not surprising. However, differential changes in different TG(O) species after SLO supplementation, and the distinctive composition of TG(O) in SLO and plasma of SLO-supplemented individuals (Supplemental Fig. 1), are suggestive of substantial remodeling of the acyl chain portion of SLO TG(O) species after ingestion. SLO supplementation also led to increases of plasma ether phospholipid subclasses PE(O) (162%), PC(O) (39%), LPC(O) (24%), and PE(P) (29%) (Fig. 5). It should be noted that a significant increase in PE(P) but not in other ether lipid classes within a particular circulatory lipoprotein class (HDL3) was also observed with statin treatment (4 mg/day) for 180 days (36); however, this increase is likely to be a nonspecific downstream effect of other metabolic events (reduction in oxidative stress or improvement in dyslipidemia).

The differential changes in different ether phospholipid classes observed with SLO supplementation can be explained by looking at the plasmalogen biosynthesis pathway (Fig. 2). PE(O) and PC(O) are formed by the addition of either ethanolamine or choline to alkyl-acyl-glycerol (37, 38). PC(O) can be deacylated by the enzyme phospholipase A2 to LPC(O); however, there is no known pathway for the direct conversion of PC(O) into PC(P), which explains the greater accumulation of LPC(O) and PC(O) in plasma after SLO supplementation. PE(O) can be converted to PE(P) by the enzyme plasmanylethanolamine Δ1-desaturase (39, 40, 41), and PE(P) can be enzymatically transformed into PC(P) (9, 42). Our findings indicate that the enzymes responsible for the conversion from PE(O) to PE(P) and PC(P) are limiting in the liver, leading to a greater proportional increase of PE(O) in plasma. We also noted that SLO supplementation predominantly increased the levels of ether phospholipids with O-18:1 alkyl chain and indeed modified the alkenyl chain composition of PE(P), with gains in O-18:1 (and O-20:1) at the expense of species containing other alkenyl chains (Fig. 7). This is due to the predominance of O-18:1 alkyl chain in SLO alkylglycerols (Fig. 4B) and the inability of these chains to be remodeled in the same way that acyl chain remodeling occurs via phospholipase and acyltransferase activities. These observations suggest that the alkylglycerol composition of dietary supplements can alter the composition of endogenous ether phospholipids and so should be considered in the formulation of future alkylglycerol supplementations.

After SLO supplementation, we also observed a decrease in PC (19%), the most abundant phospholipid in circulating lipoproteins. This was partially offset by the corresponding increases in the PC and PE ether lipids (PC(O) and PE(O)) but also reflected an overall decrease in plasma lipids. There were concomitant decreasing trends in plasma ceramide (16%), sphingomyelin (7%), PE (25%), phosphatidylinositol (26%), lysophosphatidylinositol (15%), phosphatidylglycerol (3%), COH (11%), cholesterol ester (15%), diacylglycerol (37%), and triacylglycerol (27%) with SLO supplementation (Fig. 5). Correcting for decreasing PC levels eliminated most of this effect, indicating that these decreases might be linked to overall lipoprotein levels, while strengthening the observed increases in the ether lipids PE(P) (59%), PC(P) (22%), PE(O) (224%), PC(O) (72%), LPC(O) (53%), and TG(O) (848%) and highlighting the proportional increases in all ether lipid classes.

Although we observed a substantial enrichment of ether lipid classes within the plasma lipidome after SLO supplementation, our data do not provide insight into the effect of SLO supplementation on individual lipoprotein classes. Moreover, SLO supplementation for a short duration (3 weeks) may be insufficient to ensure a steady state in the lipidome of plasma lipoproteins. Hence, further in-depth and time course studies on the impact of SLO supplementation on individual lipoprotein classes are warranted.

### SLO supplementation impacts the white blood cell lipidome

In addition to the changes in plasma lipids, we also observed increases in the levels of plasmalogens (PC(P) (31%) and PE(P) (15%)) as well as intermediates of the plasmalogen biosynthetic pathway (PC(O) (61%) and PE(O) (28%)) in circulating white blood cells upon SLO supplementation. This is important as it demonstrates that the supplemented SLO alkylglycerols are being incorporated into cells and tissues, where they may influence cell/tissue function. However, further studies to assess the impact of the enrichment of immune cell plasmalogens after SLO supplementation on immune cell function are warranted. Indeed, plasmalogens have been reported to provide functional benefits to immune cells. In vitro studies demonstrate that enrichment of RAW macrophages with plasmalogens increases cellular resistance to chemical hypoxia and reactive oxygen species (11) and enhances their phagocytic activity (43). Moreover, synthetic analogues of lyso PE(P) have been found to be highly potent in activating thymic and peripheral invariant natural killer T cells (cells with important immunoregulatory functions) (44), suggesting potential immunomodulatory functions of plasmalogens. Furthermore, during in vitro differentiation of human monocytes to macrophages, the plasmalogen profiles have been found to change, suggesting a dynamic role of plasmalogens in preparing these cells for their phagocytic and inflammatory roles (15).

### SLO supplementation has beneficial effects on clinical lipids and inflammatory markers

In addition to the changes in plasma and white blood cell molecular lipid species, SLO supplementation was also found to reduce the plasma levels of free COH and triglycerides, and the inflammatory marker, hsCRP. Our observation of decreased COH is also supported by a previous study showing the COH-lowering effect of SLO alkylglycerols in obese individuals (45). The plasma triglyceride–lowering effect of SLO was not reported before. It is beyond the scope of this study to elucidate the mechanism behind this effect. Moreover, we cannot exclude the possibility of driving triglyceride accumulation in the liver by SLO supplementation and that as the major contributor of lower triglyceride in the plasma.

Here, we also observed a significant decrease in hsCRP after SLO supplementation. CRP is regarded as prothrombotic and proatherogenic in nature and is commonly used as a marker of systemic inflammation (46). Similar reductions in hsCRP levels in old-aged surgical patients with SLO supplementation have also been reported (28). Another study observed reduced serum complement (C3 and C4) and plasma vascular endothelial growth factor levels with SLO supplementation in obese individuals (45). Altogether, supplementation of SLO seems to have a role in modifying systemic inflammation in humans and could be effective in reducing the risk of progression of metabolic diseases to more advanced forms.

In this study, we did not observe any detrimental effect of SLO supplementation in humans. The SLO that we used for this study (Alkyrol®) has been purified to enrich for alkylglycerols and to remove all contaminants and unwanted substances such as polychlorinated biphenyl, pesticides, heavy metals, fatty substances such as COH, squalene, and excessive amounts of vitamin A and D. However, nonpurified or semipurified SLO could have deleterious effects on the liver and other major organs because of the presence of substantial amount of squalene and other undesirable components. Therefore, SLO should be consumed with caution. There are some alternatives such as monoalkylglycerols (chimyl alcohol, batyl alcohol, or selachyl alcohol) (47) or marine food supplements (48), which can be used to enrich endogenous plasmalogens. However, the currently available mono-alkylglycerols are not approved for human consumption, and the potential of marine food supplements in modulating endogenous plasmalogens is still in question.

## Conclusion

SLO supplementation modulated plasmalogens and other ether lipids in human plasma and white blood cells. These changes, together with the observation of small but significant improvements in clinically important markers of dyslipidemia and inflammation, provide a strong rationale for larger trials examining the impact of SLO supplementation on metabolic diseases.

## Data availability

All data in this article are contained within this article and are available upon request to the corresponding author.

## Supplemental data

This article contains supplemental data.

## Conflict of interest

The authors declare that they have no conflicts of interest with the contents of this article.
