# Supplementary material for: Shark liver oil supplementation enriches endogenous plasmalogens and reduces markers of dyslipidemia and inflammation
Source: J Lipid Res. 2021 Jun 17;62:100092. doi: 10.1016/j.jlr.2021.100092 (PMC8281607; doi:10.1016/j.jlr.2021.100092)
Supplement: Tables S1–S8 [file mmc2.docx]

**Supplemental Figures:**

**Fig. S1. Plasma ether lipid levels in the pre-SLO and pre-Placebo groups.**

*Date are presented as mean ± SEM; SLO: shark liver oil; PC(O): alkylphosphatidylcholine; PC(P): alkenylphosphatidylcholine; LPC(O): lysoalkylphosphatidylcholine; PE(O): alkylphosphatidyl-ethanolamine; PE(P): alkenylphosphatidyl-ethanolamine; TG(O): monoalkyl-diacylglycerol.*

**Fig. S2. Composition of monoalkyl-diacylglycerol ((TG(O) [SIM]) in shark liver oil and plasma of the participants before (pre-SLO) and after (post-SLO) shark liver oil supplementation.**

**Fig. S3. Effect of shark liver oil supplementation on plasma lipid concentrations normalised to phosphatidylcholine concentration.**

*Estimated effects of shark liver oil (SLO) supplementation on (log-transformed) plasma lipid concentrations (normalised to phosphatidylcholine concentration) relative to placebo treatments. Open grey circles: lipid species, non-significant, no confidence intervals (CIs); violet circles: lipid species, nominally significant (P < 0.05), with CIs; blue circles: lipid species, significant after multiple testing correction (P < 0.05) using Benjamini-Hochberg’s approach, with CIs; red diamonds: lipid class/sub-class totals, significant after multiple testing correction (P < 0.05) using Benjamini-Hochberg’s approach, with CIs. dhCer: dihydroceramide; Cer: ceramide; HexCer: monohexosylceramide; Hex2Cer: dihexosylceramide; Hex3Cer: trihexosylceramide; GM3: G_M3_ ganglioside; SM: sphingomyelin; PC(O): alkylphosphatidylcholine; PC(P): alkenylphosphatidylcholine; LPC: lysophosphatidylcholine, LPC(O): lysoalkylphosphatidylcholine; PE: phosphatidylethanolamine; PE(O): alkylphosphatidylethanolamine; PE(P): alkenylphosphatidylethanolamine; LPE: lysophosphatidylethanolamine; PI: phosphatidylinositol; LPI: lysophosphatidylinositol; PS: phosphatidylserine; PG: phosphatidylglycerol; COH: cholesterol; CE: cholesteryl ester; DG: diacylglycerol; TG: triacylglycerol; TG(O): TG(O) [NL], monoalkyl-diacylglycerol.*
